# Kawain Inhibits Urinary Bladder Carcinogenesis through Epigenetic Inhibition of LSD1 and Upregulation of H3K4 Methylation

**DOI:** 10.3390/biom13030521

**Published:** 2023-03-13

**Authors:** Xia Xu, Xuejiao Tian, Liankun Song, Jun Xie, Joseph C. Liao, Joshua J. Meeks, Xue-Ru Wu, Greg E. Gin, Beverly Wang, Edward Uchio, Xiaolin Zi

**Affiliations:** 1 Department of Urology, University of California, Irvine, Orange, CA 92868, USA; 2 Veterans Affairs Long Beach Healthcare System, Long Beach, CA 90822, USA; 3 Veterans Affairs Palo Alto Health Care System, 3801 Miranda Avenue, Palo Alto, CA 94304, USA; 4 Jesse Brown VA Medical Center, 820 S Damen Ave, Chicago, IL 60612, USA; 5 Veterans Affairs New York Harbor Healthcare System, New York, NY 10010, USA; 6 Department of Pathology and Laboratory Medicine, University of California, Irvine, CA 92868, USA; 7 Chao Family Comprehensive Cancer Center, University of California, Irvine, CA 92868, USA

**Keywords:** kava, LSD1, chemoprevention, bladder cancer

## Abstract

Epidemiological evidence suggests that kava (*Piper methysticum Forst*) drinks may reduce the risk of cancer in South Pacific Island smokers. However, little is known about the anti-carcinogenic effects of kava on tobacco smoking-related bladder cancer and its underlying mechanisms. Here we show that dietary feeding of kawain (a major active component in kava root extracts) to mice either before or after hydroxy butyl(butyl) nitrosamine (OH-BBN) carcinogen exposure slows down urinary bladder carcinogenesis and prolongs the survival of the OH-BBN-exposed mice. OH-BBN-induced bladder tumors exhibit significantly increased expression of lysine-specific demethylase 1 (LSD1), accompanied by decreased levels of H3K4 mono-methylation compared to normal bladder epithelium, whereas dietary kawain reverses the effects of OH-BBN on H3K4 mono-methylation. Human bladder cancer tumor tissues at different pathological grades also show significantly increased expression of LSD1 and decreased levels of H3K4 mono-methylation compared to normal urothelium. In addition, kava root extracts and the kavalactones kawain and methysticin all increase the levels of H3K4 mono- and di-methylation, leading to inhibitory effects on cell migration. Taken together, our results suggest that modification of histone lysine methylation may represent a new approach to bladder cancer prevention and treatment and that kavalactones may be promising agents for bladder cancer interception in both current and former smokers.

## 1. Introduction

Human urinary bladder cancer (BCa) is the fourth most common cancer, with a lifetime risk of 1 in 27 among American men [1]. Bladder cancer occurs primarily in aging men with a history of cigarette smoking. Cigarette smoking has been estimated to be a contributing factor in 40–70% of all BCa cases in the United States (US) [2]. Smoking is the most modifiable risk factor for bladder cancer. The cessation of smoking leads to a reduction in the incidence of bladder cancer and has thus become a primary focus for reducing the incidence of bladder cancer [3]. However, although the prevalence of cigarette smoking in the US has substantially decreased, falling from 42% to 20.8% of adults during the last 30 years, incidence rates of bladder cancer have remained stable in the US in men (123.8 to 142.2/100,000 person-years) and women (32.5 to 33.2/100,000 person-years) during the same period [4]. A recent study [5] has reported an increased risk of bladder cancer associated with cigarette smoking over time, suggesting a stronger association between cigarette smoking and bladder cancer at present than in the past. Stronger associations between smoking and bladder cancer could potentially offset the decreased prevalence of smoking in the US population and contribute to the stability of bladder cancer incidence rates. Moreover, of all the smoking-related cancers, bladder cancer has the highest prevalence, even higher than lung cancer [6]. Therefore, in addition to smoking cessation, new strategies should be developed for bladder cancer prevention in former and current smokers.

Recent evidence indicates that modification of reversible epigenetic events, such as histone acetylation and methylation during carcinogenesis, may be one of the most promising ways to prevent tobacco-smoking-related cancers [7,8,9,10,11]. Kava has been an ancient drink in the Pacific Islands for thousands of years [12]. According to the 2018 GLOBOCAN’s Cancer Incidence and Mortality Worldwide report, age-standardized incidences of tobacco-smoking-related lung and bladder cancers in three kava-drinking Pacific countries (Fiji, Vanuatu, and Samoa) were markedly lower, despite the high percentages of smokers (up to 58.3% of men in Samoa), than those in the neighboring countries, such as Australia and New Zealand [13,14,15]. In addition, kava root extracts have recently been found to exhibit potent anti-cancer and anti-carcinogenic activities in several cancers, including lung, prostate, and colon cancers [16,17,18,19]. We have recently reported that kava root extracts and kavalactones, including kawain, are cell-active and weak inhibitors of monoamine oxidase A (MAO-A) and lysine-specific demethylase 1 (LSD1) enzymes [17]. LSD1 has been suggested to be a key therapeutic target in various cancers via its demethylase activity against both histone (i.e., mono- and di-methylated histone 3, lysines 4 and 9), and non-histone targets (e.g., FOXA1), and several LSD1 inhibitors are currently under different stages of clinical trials for the treatment of cancer [20,21]. Kawain is a major kavalactone from kava root extracts [22]. However, the role of LSD1 in bladder carcinogenesis remains unknown. In addition, there are very few studies on the anti-tumor effects of kawain as a pure component, and no animal experimental study of the chemopreventive properties of kawain against tobacco-smoking-related human bladder cancer has been reported.

In the present study, we have examined the chemopreventive efficacy of the kawain diet on tumor growth and progression in the OH-BBN–induced bladder cancer model, which was administered not only in the pre-initiation phase but also in the post-initiation phase, continually throughout the full course of carcinogenesis. Additionally, we have examined the potential of LSD1 as a molecular target for the chemopreventive effects of kawain against urinary bladder carcinogenesis in the OH-BBN–induced bladder cancer model.

## 2. Materials and Methods

### 2.1. Chemopreventive Effects of Dietary Kawain on Urothelial Carcinogenesis Induced by Tobacco Smoke Carcinogen OH-BBN-Induced Bladder Cancer

**Figure 1:**
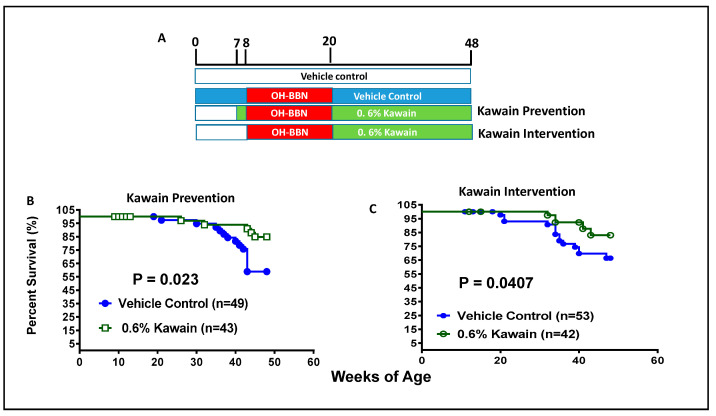
Dietary feeding of kawain inhibits OH-BBN-induced bladder carcinogenesis in mice. (**A**) A schematic presentation of experimental protocols for the anti-bladder carcinogenic effect of the kawain diet before and after OH-BBN exposures. (**B**,**C**) The survival curves of mice that were administrated with a control diet or with 0.6% kawain in the diet starting one week prior to OH-BBN administration or starting post-OH-BBN administration.

Male B6D2F1 (C57B1/6 × DBA/2 F1) mice (Harlen-Sprague Dawley), four weeks old on arrival, were fed the AIN-76A semi-purified diet. The standard procedure to produce the OH-BBN-induced bladder carcinogenesis model was followed as described previously [23]. Each mouse at eight weeks of age received 7.5 mg of OH-BBN (TCI America, Portland, OR, USA) by gavage once per week for twelve weeks. Mice were assigned to experimental groups using a randomization process designed to ensure a comparable initial body weight in each group, and the experiment ended 40 weeks after the initial OH-BBN treatment. Kawain was administered as dietary supplementation to mice beginning either one week before the first OH-BBN administration (prevention protocol) or one week after the final OH-BBN administration (week 1 or 13) (intervention protocol), as shown in Figure 1A. A dietary control group receives a vehicle diet without added kawain. In the prevention or anti-initiation protocol, the mice were randomly assigned to either a control diet or one with 0.6% kawain in the diet at seven weeks of age before OH-BBN exposure. In the intervention or anti-promotion protocol, the control diet and a 0.6% kawain diet were given to the mice at 20 weeks of age after OH-BBN exposure. The mice were continuously fed with these experimental diets until they reached 48 weeks of age or died due to the development of urothelial tumors (Figure 1A). Food consumption, body weight, and health conditions of the mice were also monitored weekly. In all groups, exposure to kawain or vehicles was continued until study termination. The mice were weighed weekly and palpated for bladder masses twice per week. The food consumption was recorded weekly. At the end of treatments, the death (free) rate, bladder weight, tumor weight, frequency of tumor formation, and tumor morphology were evaluated by pathological and statistical analyses.

### 2.2. Necropsy, Histological, and Immunohistochemical (IHC) Examination

Necropsy notes were collected on all animals. The notes included quantitative and qualitative descriptions of the bladder, lymph nodes, and tissues showing any visible abnormalities. Photographs were taken by a digital camera to document the animals and their urogenital systems in situ. Bladders were removed and inflated with 10% buffered formalin. The fixed bladders were longitudinally bisected and further transversely cut into six pieces. The gross lesions in these pieces were observed under microscopy. Histological sections were analyzed with hematoxylin and eosin (H&E) staining. Sample slides were sent for pathological analysis, and were assigned special codes to avoid observer bias.

In addition, slides of bladder tumors were stained by mouse monoclonal anti-histone H3 monomethyl Lysine 4 (H3K4-me) (Abcam, 1:400), anti-LSD1 (Abcam, 1:500), anti-Ki-67 antibodies (Abcam, 1:800), and appropriate secondary antibodies for IHC analysis of cell proliferation, as described previously [23,24,25]. Negative controls were treated only with PBS under identical conditions. Positive staining cells were quantified double-blinded by tallying the total number of cells in 12 arbitrarily selected fields at ×200 magnification. The staining intensity is scored as 0, 1, 2, or 3, which correspond to negative, weak, moderate, or strong staining intensities, respectively. Percentages of stained cells are determined by counting at least 100 cells, and a final H-score is calculated as the product of staining intensities (0–3) and percentages of stained cells (0–100%).

### 2.3. Cell Migration

The T24 cell line was obtained from the American Type Culture Collection (ATCC) (Manassas, VA) and cultured in a McCoy 5A growth medium enriched with 10% fetal bovine serum (FBS). T24 cells were tested for known species of mycoplasma contamination using a kit from Lonza, Inc. A total of 1 × 10^6^ cells were plated in six wells in a growth medium until they reached a confluence of approximately 90%. Cell migration was assessed with a scratch assay [26]. A scratch was made through each well using a sterile pipette tip. The monolayer was incubated with a migration assay buffer consisting of serum-free medium and different concentrations of kawain from LKT Laboratories, Inc. (St. Paul, MN, USA) and LSD1 inhibitor II from Sigma-Aldrich, Inc. (St. Louis, MO, USA). Images were captured at the same position at 0 and 16 h. The area of the wound that healed was calculated with ImageJ software [27].

### 2.4. Western Blotting Analysis

Cell lines 5637 and T24 were obtained from ATCC (Manassas, VA, USA) and cultured in an RPMI growth medium enriched with 10% FBS. Kava root extract (KRE) at a concentration of 150 mg/mL kavalactones in 50% ethanol was obtained from Gaia Herbs (Brevard, NC, USA). Pure kawain and methysticin (99%) were purchased from LKT Laboratories, Inc. (St. Paul, MN, USA). LSD1 inhibitor II and 2-tranylcypromine (2-PCPA) were from Selleck Chemicals LLC (Houston, TX, USA). 5637 and T24 cells were tested for known species of mycoplasma contamination using a kit from Lonza, Inc. The 5637 and T24 cells were treated with 0.1% DMSO and different doses or concentrations of KRE, kawain, and methysticin for 24 h. After treatments, cells were lysed in a radio-immunoprecipitation assay (RIPA) buffer containing a protease inhibitor cocktail. After centrifugation, supernatants were collected and quantified for protein concentrations by the Bio-Rad protein assay as described previously [28]. Clarified protein lysates (60–100 µg) were denatured in 2x loading buffer at 100 °C and then resolved on 8–16% SDS-PAGE. Proteins were transferred to nitrocellulose membranes, probed with indicated antibodies, and visualized by an enhanced chemiluminescence detection system. β-tubulin was used as a loading control.

### 2.5. Statistical Evaluation

Data were presented as means ± SD. Statistical analyses were performed using the analysis of variance (ANOVA). Welch’s two-sample t-tests were used to determine whether the means of the two populations were different. *p*-value: evidence that the means are different (*p* ≤ 0.05 was taken as significant). Statistical analyses were performed by SPSS 17.0 and Excel. Kaplan-Meier survival curves were plotted and analyzed by the log-rank test in the Prism8 software.

## 3. Results

### 3.1. Administration of a Kawain-Formulated Diet before and after Tobacco Carcinogen OH-BBN Exposures Increases the Survival of Mice

Figure 1B shows that 84.9% of mice in the kawain diet group and 58.9% of mice in the control diet group survive beyond 48 weeks of age. The kawain diet increased the percentage of surviving mice by an absolute 26% in the prevention protocol (*p* = 0.023, Figure 1B). In the intervention protocol, the kawain diet increases the percentage of surviving mice over 48 weeks of age by an absolute 16.6% (*p* = 0.0407, Figure 1C). These results indicate that dietary kawain has both anti-initiation and anti-promotion effects against OH-BBN-induced urothelial tumorigenesis. 

### 3.2. Kawain Diet Reduces the Weight of OH-BBN-Induced Bladder Tumors in Both Prevention and Intervention Protocols

**Figure 2:**
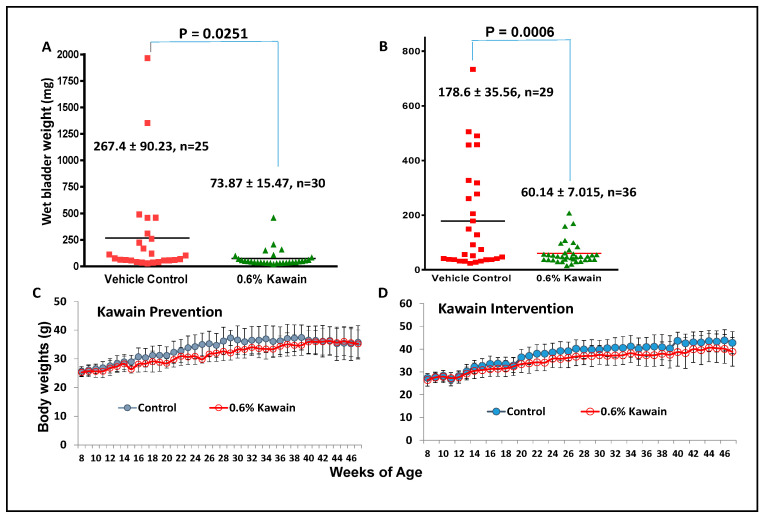
Dietary feeding of kawain alleviates tumor burden in the OH-BBN-induced mouse bladder carcinogenesis model. (**A**,**B**) The mean bladder weights of the mice treated with the control diet or 0.6% kawain diet at the end of experiments in the prevention and intervention protocols, respectively. (**C**,**D**) The mean body weights of the mice treated with the control diet or 0.6% kawain diet over time in the prevention and intervention protocols, respectively. Error bars are means of body weights ± standard deviations.

The mean wet bladder weights of surviving mice at the end of the experiment in the kawain diet group as a surrogate for tumor burden were reduced by 72.4% and 66.3% in the prevention and intervention protocols, respectively, compared to those of mice in the control diet group (Figure 2A,B, Ps = 0.0251 and 0.0006, respectively, in the prevention and intervention protocols). The mean body weights of the mice over time in the kawain diet group also appear to be slightly lower than those of the mice in the control diet group, but the differences are not statistically significant (Figure 2C,D). There are no significant differences in food consumption between the two groups (data not shown). 

### 3.3. Kawain Diet Decreases the Incidences of OH-BBN–Induced Muscle-Invasive Urothelial Tumors in Mice

**Figure 3:**
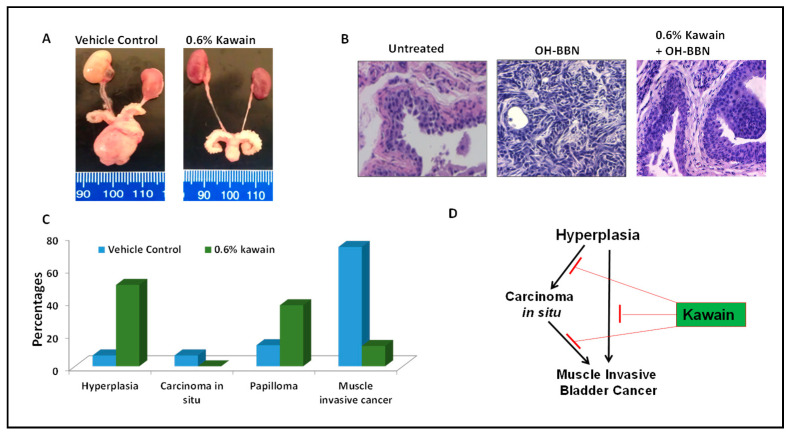
Dietary feeding of kawain inhibits the progression of hyperplasia to carcinoma in situ and muscle-invasive bladder tumors. (**A**) A representative photograph of representative genitourinary systems from mice fed with a vehicle diet or a diet supplemented with 0.6% kawain. (**B**) Microscopic examination of H&E-stained bladder tissues from mice treated with the control diet, OH-BBN only, and OH-BBN plus the kawain diet. Magnification: 200×. (**C**) Percentages of hyperplasia, carcinoma in situ, papilloma, and muscle-invasive cancer in mice exposed to OH-BBN and treated with a vehicle control diet or a diet supplemented with 0.6% kawain. (**D**) Schematic presentation of the effect of the kawain diet on the pathological progression of OH-BBN-induced urinary bladder cancer in mice.

Macroscopic examination revealed that OH-BBN induced the enlargement of kidneys (or hydronephrosis), ureters, and bladders in mice from the control diet group, compared to those from the kawain diet group (Figure 3A). H&E staining and histological examination classified them as: (1) normal urothelial epithelium, featured by the epithelium of fewer than three layers without any anaplasia; (2) urothelial dysplasia, featured by the epithelium of three or more layers with moderate to severe anaplasia with diffused proliferation; (3) carcinoma in situ, featured by thickened urothelium and profound nuclear atypia, crowding, a high nuclear/cytoplasmic ratio, frequent mitotic figures, and loss of cellular polarity; and (4) invasive urothelial carcinoma, characterized by the invasion of the submucosa or muscle layer with squamous differentiation features (Figure 3B). Administration of 7.5 mg of OH-BBN once per week for 12 weeks induced 6.7% of mice at the age of 48 weeks with urothelial dysplasia, 6.7% carcinoma in situ, 13% papillomas, and 73.3% invasive urothelial carcinoma mixed with squamous differentiation features. The kawain diet given before OH-BBN until the end of the experiment resulted in a significant reduction in the incidence of invasive urothelial carcinoma by 60.8% (*p* < 0.01). No in situ carcinoma lesions were detected in the kawain diet group. These data imply that the kawain diet impedes tumor progression at early and non-invasive stages of bladder cancer with a marked reduction in carcinoma in situ (a precursor to invasive bladder cancer) and invasive urothelial cell carcinoma in OH-BBN–induced urinary bladder tumorigenesis.

### 3.4. LSD1 Is Overexpressed and H3K4me Level Is Downregulated in Both Human and Mouse Bladder Tumor Tissues Compared to Normal Bladder Urothelium

**Figure 4:**
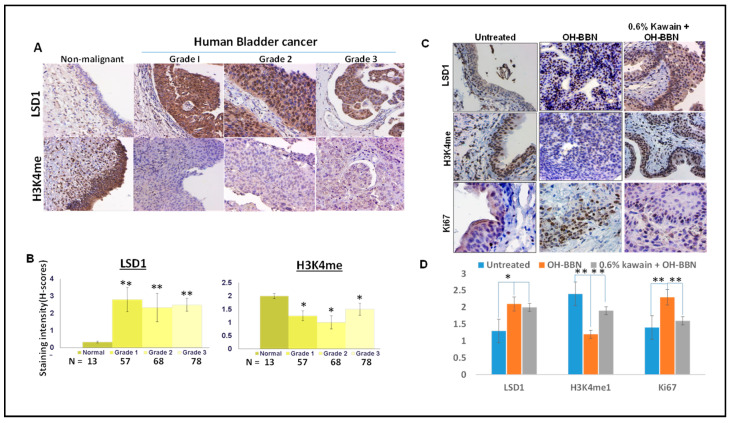
LSD1 is overexpressed and H3K4me level is downregulated in both human and mouse bladder cancer tissues compared to normal bladder urothelium. (**A**) LSD1 and H3K4 me expressions were examined by IHC in human normal bladder tissues and different grades of bladder cancer tissues. Magnification ×200. (**B**) The staining intensity is scored as 0, 1, 2, or 3, which correspond to negative, weak, moderate, or strong staining intensities, respectively. Percentages of stained cells are determined by counting at least 100 cells, and a final H-score is calculated as the product of staining intensities (0–3) and percentages of stained cells (0–100%). (**C**) LSD1, H3K4me, and Ki-67 expressions were examined by IHC in bladder tissues from mice treated with a control diet, OH-BBN only, and OH-BBN plus the kawain diet. Magnification ×200. (**D**) Quantitative analysis of staining intensities (H-score). Error bars are standard errors. “*” denotes *p* < 0.05 and “**” *p* < 0.01.

IHC analysis was used to examine expression levels of LSD1 and the methylation status of H3K4 in commercial tissue microarrays (TMA) containing 203 tissue samples from different stages of bladder cancer and 13 normal bladder tissue controls (Biomatrix, LLC, Plantation, FL, USA). Figure 4A,B show that LSD1 levels were equally elevated and mono-methylation levels of H3K4 were down-regulated in all three different grades of bladder tumors compared with normal tissues (Ps < 0.001, Mann-Whitney’s U-test). Strong LSD1 staining was detected mainly in the nucleus of malignant cells compared to non-malignant cells. LSD1 staining levels were inversely related to mono-methylation levels of H3K4. These results suggest that LSD1 overexpression may be involved in the early or initial stage of urinary bladder carcinogenesis. Similarly, Figure 4B,D show the absence of LSD1 staining and strong H3K4me staining in the nuclei of the normal urothelium from untreated mice, whereas OH-BBN treatment significantly increased the expression of LSD1 and decreased the expression of H3K4me in mouse bladder tumor tissues. In addition, the kawain diet reversed the effect of OH-BBN on the expression of H3K4me and reduced the expression of the cell proliferative marker Ki67 (Ps < 0.001, Mann-Whitney’s U-test, Figure 4B,D). These results imply a novel mechanism of kawain’s action via regulating LSD1 activity in vivo in the OH-BBN model for cancer prevention and intervention.

### 3.5. Kava Root Extracts and Kavalactones Increase the Expression of H3K4me and H3K4me2 in Bladder Cancer Cell Lines

**Figure 5:**
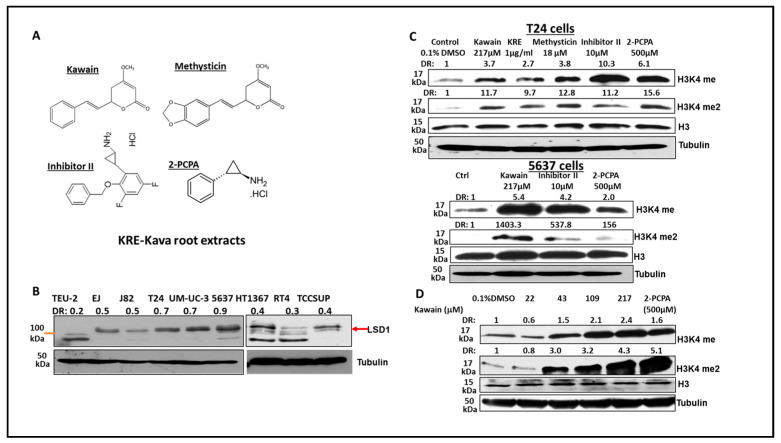
Kava root extracts, kawain, and methysticin induce mono- and di-methylation of H3K4 in bladder cancer cell lines. (**A**) Chemical structures of kawain, methysticin, 2-PCPA, and inhibitor II. Bladder cancer cells were treated for 24 h with the indicated concentrations of kawain, kava root extracts (KRE), and methysticin. (**B**) Western blotting analysis of LSD1 expression in non-malignant bladder epithelial TEU-2 cells and bladder cancer cell lines. (**C**) Western blotting analysis was performed to examine the methylation status of H3K4 in T24 cells and 5637 cells using specific anti-H3K4me and me2 antibodies (Abcam). Histone H3 and tubulin were used as internal controls. A representative blot from three independent experiments was shown. (**D**) T24 cells were treated with different concentrations of kawain and 500 μM 2-PCPA for 24 h. Expression of H3K4me and H3K4me2 was examined similarly, as described above.

LSD1 shares significant similarities in its catalytic domains with other members of the amine oxidase family. As such, monoamine oxidase inhibitors, such as 2-PCPA, have been used as non-selective LSD1 inhibitors to treat cancer [28,29]. We have recently demonstrated the inhibitory effects of kava root extracts and kavalactones in prostate cancer cell lines on MAOA and LSD1 enzymes [17]. LSD1 is an enzyme that can remove mono- and di-methylated H3K4. Figure 5A shows the chemical structures of 2-PCPA, LSD1-specific inhibitor II, kawain, and methysticin. Figure 5B shows that LSD1 is overexpressed in all bladder cancer cell lines, which were derived from superficial bladder tumors and different stages (T2–T4) of muscle-invasive bladder tumors. At a concentration of 217 micromolars, kawain effectively induced the expression of H3K4me and HeK4me2, similar to the effects of 10 micromolar of LSD1-specific inhibitor II or 500 micromolar of 2-PCPA in bladder cancer cell lines (Figure 5C). Kava root extracts and methysticin showed more potent effects on the elevation of H3K4me and H3K4me2 protein levels (Figure 5C). Kawain induced the expression of H3K4me and HeK4me2 in a dose-dependent manner (Figure 5D). These results further confirmed that kawain is a weak and cell-active inhibitor of LSD1. 

**Figure 6:**
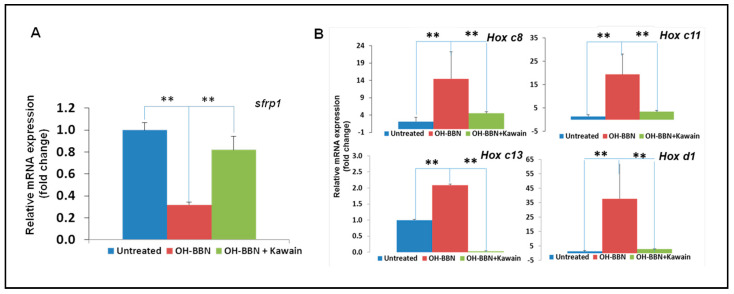
The kawain diet reverses the effects of OH-BBN on the expression of LSD1 target genes. (**A**) RNAs were extracted from bladder tissues of untreated, exposed to OH-BBN only, or OH-BBN plus the kawain diet mice. (**B**) Real-time RT-PCR was performed to analyze the mRNA expression of LSD1 target genes (PSA and TMPRSS2). Bars are the means ± SD of three independent experiments. The differences in mRNA expression levels were compared between OH-BBN only treatment and untreated or OH-BBN plus kawain-treated mice (*n* = 6 in each group). “**” denotes *p* < 0.01.

In addition, we found that OH-BBN treatment increased the expression of some LSD1 target genes, including *sFRP1*, *Hox c8*, *c11*, and *c13*, and *Hox d1*, and that the kawain diet reversed the effects of OH-BBN on the expression of LSD1 targeted genes in bladder tumor tissues of the OH-BBN bladder carcinogenesis model (Figure 6). 

### 3.6. Kawain Inhibits the Migration of Bladder Cancer Cell Line T24

**Figure 7:**
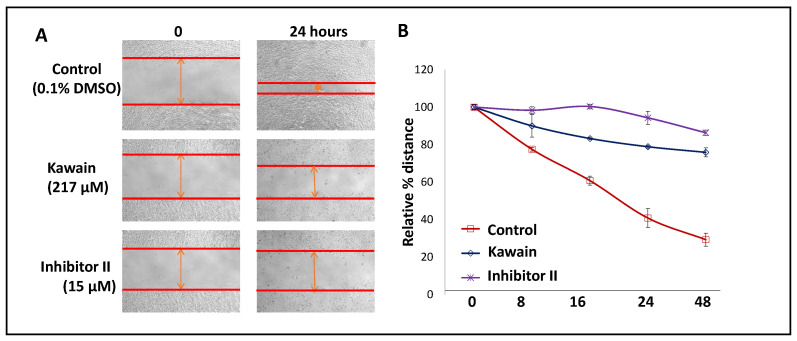
Kawain inhibits cell migration. (**A**) Microphotographs show repopulation of wounded areas of vehicle control (0.5% DMSO) or kawain or inhibitor II treated T24 cells. (**B**) Line graphs of widths of the gap with indicated treatments at 0, 8, 16, 24, and 48 h. Points are percentages of the widths of the gap related to control at 0 h.

We examined, by using a wound-healing assay, the effect of kawain on the migration of T24 cells. Figure 7A,B show that kawain and LSD1 inhibitor II treatment resulted in about 3.5- and 5.5-fold slower migration into the wounded area, measured by the width of the gap, respectively, when compared with vehicle control treatment (Ps < 0.01).

## 4. Discussion

About 75% of human bladder cancers are associated with cigarette smoking or exposure to industrial carcinogens [2]. The most common carcinogens in cigarette smoking and industrial exposure are aromatic amines and nitrosamines that have been detected in human urine [30]. Accordingly, the mouse or rat model of urinary bladder cancer induced by the organ-specific nitrosamine OH-BBN has been established [31]. The OH-BBN-induced bladder tumors in mice express basal subtype cell markers (i.e., *Cd44, Cdh3, Krt5,* and *Krt14*) with highly frequent mutations (70 to 90%) of *Trp53* and H3K4 methyltransferase family members, including lysine methyltransferase 2C (*Kmt2c*) and *Kmt2d*, which closely mimic the basal/squamous type of human muscle-invasive bladder cancer, both molecularly and pathologically [7,8,9]. Therefore, this model is a suitable one for studying the chemoprevention of tobacco-smoking-related bladder cancers through epigenetic approaches. There are very few studies on the anti-carcinogenic effects of kawain as a pure component, and no animal experimental studies of the chemopreventive properties of kawain toward tobacco carcinogen OH-BBN-induced urinary bladder cancer have been reported [24,32,33]. Here, we have shown that dietary kawain significantly extended the survival of mice with OH-BBN-induced bladder tumors and reduced tumor growth both in the prevention protocol (kawain diet was given before carcinogen OH-BBN administration) and the intervention protocol (kawain diet was given before carcinogen OH-BBN administration). In addition, we have demonstrated that the kawain diet effectively inhibits OH-BBN-initiated urinary bladder carcinogenesis by reducing the incidence of muscle-invasive bladder tumors. 

In a chemoprevention study of tobacco smoking-related lung cancer, kava and dihydro-methysticin (DHM) have been shown to completely block the tobacco carcinogen 4-(methylnitrosamino)-1-(3-pyridyl)-1-butanone (NNK)-induced lung adenomas in A/J mice when given before administration of NNK, whereas post-NNK administration of kava and DHM had little effect on preventing lung tumorigenesis [18]. The studies further showed that kava and DHM decreased the NNK-induced formation of the DNA adduct O6-methylguanine in the lung of A/J mice and increased the urinary excretion of NNK detoxification metabolites [18,34]. In our studies, the chemopreventive effects of the kawain diet on the OH-BBN are effective in both pre- and post-OH-BBN administration of the kawain diet and appear to be less selective compared to reported studies [18,34]. Therefore, our results clearly suggest the potential use of kawain for human bladder cancer in both current and former smokers.

Unlike patients with lung cancer, whose smoking status is associated with specific mutations (such as high levels of C > A nucleotide transversions and KRAS mutations), the mutation burden is similar in bladder tumors of nonsmokers and current smokers, and there is a lack of an association between the smoking status and the mutational spectrum [35,36,37]. Instead, analysis of the Cancer Genome Atlas (TCGA) of bladder cancer data revealed that the CpG island methylator phenotype existed in 34% of bladder tumors and was positively associated with pack-years of smoking [35]. These results suggest that there may be a different mechanism of tobacco-induced carcinogenesis between bladder and lung cancers. Epigenetic mechanisms are increasingly appreciated as cell-fate-determining drivers of tobacco-related bladder cancer. We have also observed that OH-BBN treatment results in increased expression of LSD1 and reduced levels of monomethylated-H3K4 in urothelial tumor tissues in mice. Consistently, LSD1 is overexpressed and monomethylated-H3K4 down-regulated in different grades of human bladder tumors compared with normal human bladder urothelium. LSD1 is a flavin-dependent monoamine oxidase for demethylating mono- and di-methylated H3K4 and plays an oncogenic role in carcinogenesis by impairing differentiation and promoting proliferation, cell motility, and invasiveness [38]. KMT2C/2D are major H3K4 mono-methyltransferases and are considered tumor suppressors, which play a critical role in regulating cell fate transition and metabolism [39,40,41]. KMT2C/2D are also highly frequently mutated in both OH-BBN-induced bladder cancer and human bladder cancer [7,8,9]. Taken together, these results indicate that both LSD1 overexpression and KMT2C/2D mutations contribute to the globally lower levels of H3K4 methylation in human bladder cancer [39,40,41,42]. Interestingly, mice with targeted inactivation of KMT2C H3K4 methylation activity selectively develop urothelial tumors [40]. Therefore, restoring the methylation levels of H3K4 in bladder cancer may be a novel approach for the chemoprevention of human urinary bladder cancer in smokers. Importantly, unlike mutant KMT2C and KMT2D, overexpression of LSD1 is subjected to pharmacological intervention and is a viable target for enhancement of H3K4 methylation for bladder cancer interception. 

In this study, we demonstrated that the kawain diet effectively restored the levels of monomethylated-H3K4 in the OH-BBN-induced bladder tumors and human urinary bladder cancer cell lines. These results further confirmed our previous reports that kawain serves as a weak cell-active LSD1 inhibitor that inhibits cell proliferation and migration [17]. We also observed that the kawain diet reversed the effects of OH-BBN on cell proliferation and expression of several LSD1 target genes, including *sfrp1, Hox c8, c11, c13*, and *d1* in bladder tumor tissues [43,44]. DNA methylation discriminates between enhancers and promoters, marked by H3K4me1 and H3K4me3, respectively: low-methylated regions are H3K4me3-enriched, while those with intermediate DNA methylation levels are progressively H3K4me1-enriched; H3K4me1 is enriched at the active and primed enhancers [45]. Transcriptional enhancers control cell-identity gene expression and thus are important for cell identity [44]. Further studies were warranted to examine whether LSD1 expression is related to a specific subtype (i.e., basal/squamous) of human urinary bladder cancer and to develop a more potent LSD1 inhibitor for bladder cancer prevention and treatment. 

## 5. Conclusions

In summary, genomic sequencing data have shown that up to 90% of human urinary tumors harbor inactivating mutations in at least one chromatin-modifying enzyme [8]. In particular, KMT2C and KMT2D mutations are very common and lead to lower levels of H3K4 mono-methylation [7,8,9]. Our results from the analysis of the OH-BBN-induced bladder tumors and cancers and normal urothelial tissue samples have suggested that restoring the levels of H3K4 mono-methylation by targeting LSD1. This could be a novel epigenetic strategy for intercepting the progression into a muscle-invasive disease in tobacco smoking-related urinary bladder cancer. The kawain diet effectively reduces tumor growth, delays the progression to muscle-invasive lesions, and increases the survival of mice bearing the OH-BBN-induced bladder tumors, which were accompanied by increased levels of H3K4 mono-methylation and decreased cell proliferation. Our studies indicated that kawain deserves further investigation as a novel LSD1 inhibitor against human urinary bladder cancer progression through modification of aberrant epigenetic events induced by tobacco smoking.

## Data Availability

Not applicable.
